# Notes from an epicenter: navigating behavioral clinical trials on autism spectrum disorder amid the COVID-19 pandemic in the Bronx

**DOI:** 10.1186/s13063-022-06635-9

**Published:** 2022-08-19

**Authors:** Alaina S. Berruti, Roseann C. Schaaf, Emily A. Jones, Elizabeth Ridgway, Rachel L. Dumont, Benjamin Leiby, Catherine Sancimino, Misung Yi, Sophie Molholm

**Affiliations:** 1 The Cognitive Neurophysiology Laboratory, Department of Pediatrics, Albert Einstein College of Medicine, Bronx, NY USA; 2 Department of Occupational Therapy, Thomas Jefferson University School of Rehabilitation Sciences, Philadelphia, PA USA; 3 Department of Psychology, Queens College and the Graduate Center, City University of New York, Queens, NY USA; 4 The Children’s Evaluation and Rehabilitation Center, Department of Pediatrics, Albert Einstein College of Medicine, Bronx, NY USA; 5 Division of Biostatistics, Thomas Jefferson University, Philadelphia, PA USA; 6 Department of Neuroscience, Albert Einstein College of Medicine, Bronx, NY USA; 7 Department of Psychiatry and Behavioral Sciences, Albert Einstein College of Medicine, Bronx, NY USA

**Keywords:** COVID-19, Autism spectrum disorder, Behavioral intervention, Sensory integration therapy, Applied behavior analysis, Randomized controlled trial

## Abstract

**Background:**

The COVID-19 pandemic impacted nearly all facets of our daily lives, and clinical research was no exception. Here, we discuss the impact of the pandemic on our ongoing, three-arm randomized controlled trial (RCT) *Sensory Integration Therapy (SIT) in Autism: Mechanisms and Effectiveness (NCT02536365)*, which investigates the immediate and sustained utility of SIT to strengthen functional daily-living skills and minimize the presence of maladaptive sensory behaviors in autistic children.

**Main text:**

In this text, we detail how we navigated the unique challenges that the pandemic brought forth between the years 2020 and 2021, including the need to rapidly adjust our study protocol, recruitment strategy, and in-person assessment battery to allow for virtual recruitment and data collection. We further detail how we triaged participants and allocated limited resources to best preserve our primary outcome measures while prioritizing the safety of our participants and study team. We specifically note the importance of open and consistent communication with all participating families throughout the pandemic in ensuring all our protocol adjustments were successfully implemented.

**Conclusions:**

Though the COVID-19 pandemic resulted in an unprecedented interruption to in-person clinical research, clinical trials have always been and will continue to be at risk for unforeseen interruptions, whether from world events or participants’ personal circumstances. By presenting our steps to preserving this RCT throughout the pandemic, we offer suggestions for successfully managing unexpected interruptions to research. Ideally, by taking these into account, future RCTs may be increasingly prepared to minimize the impact of these potential interruptions to research.

## Background

The prolonged COVID-19 pandemic has impacted nearly all facets of our daily lives since its emergence, and clinical research has been no exception [1]. Here, we describe how our ongoing, three-arm randomized controlled trial (RCT) *Sensory Integration Therapy in Autism: Mechanisms and Effectiveness* navigated the evolving landscape of the COVID-19 pandemic between the years 2020 and 2021 (NCT02536365).

Though the phenotype of autism spectrum disorder (ASD) is heterogeneous to some degree, difficulties in processing and integrating sensory information have been reported in approximately 45–95% of autistic individuals [2]. These sensory features impact successful participation in daily living, learning, and social activities as well as behavior [3, 4], making this an important area for intervention. Occupational Therapy (OT) using Ayres Sensory Integration® (SIT) is an evidence-based intervention that is designed to address these sensory challenges and improve adaptive behaviors and participation in activities and tasks [5]. As such, our RCT was designed to improve the understanding of the immediate and sustained utility of SIT as a means of strengthening functional daily-living skills and minimizing the presence of maladaptive sensory behaviors. We aim to compare SIT mechanisms and effectiveness to that of the applied behavior analysis (ABA) approach, which has long been the standard of care for autistic children in need of support in these areas [6].

**Fig. 1:**
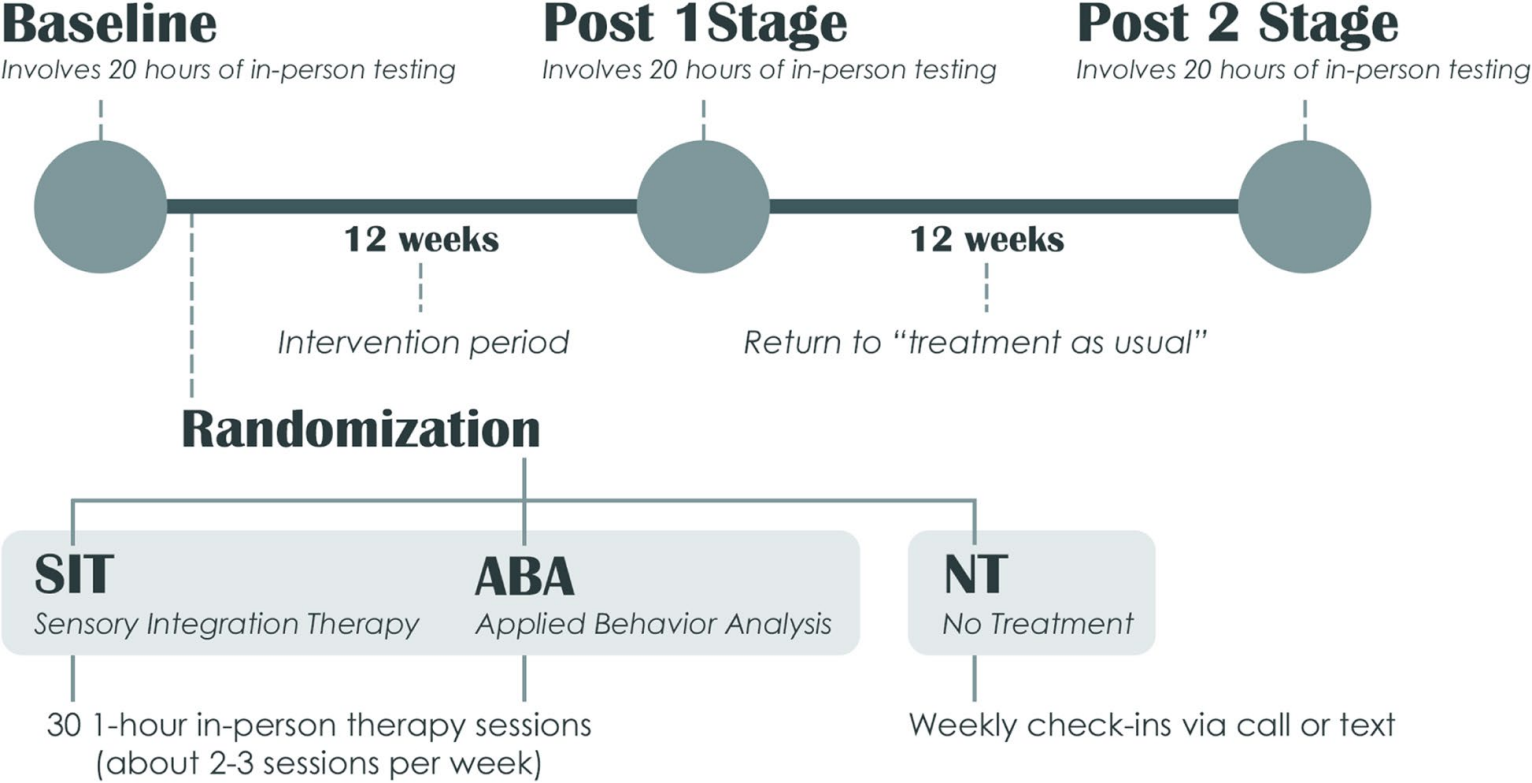
Flow of participants through RCT

To answer this question at both the behavioral and neurophysiological levels, we use an extensive battery of standardized clinical and OT assessments, parent questionnaires and interviews, and electroencephalography (EEG) recordings designed to evaluate multiple facets of our participants’ functional, social, and sensory integration skills. We recruit autistic children between 6 and 9 and a half years old who (1) demonstrate sensory dysfunction (as indexed by performance on the Sensory Integration and Praxis Test (SIPT) [7] and the Sensory Processing Measure (SPM) [8] at baseline), (2) have a nonverbal IQ of 70 or above (as indexed by performance on the Wechsler Abbreviated Scale of Intelligence–Second Edition (WASI-II) [9] at baseline), and (3) are receiving less than 12 h per week of behavioral, speech, or related therapies outside of those included in their educational programming. Participants are first assessed by a team of research-reliable psychologists, occupational therapists, and EEG technicians over the course of three to four appointments (totaling roughly 15 h of in-person testing) to establish baseline before being randomized to one of the three study arms: sensory integration therapy (SIT), applied behavior analysis (ABA), or no treatment (NT). Both SIT and ABA participants are provided 30 1-h therapy sessions across 12 weeks, while the NT participants are asked to continue their usual treatment course outside of the RCT but do not receive any new therapies. Participants are reassessed immediately (Post 1) following the 12-week intervention period and then again 12 weeks after that, during what we refer to as Post 2 [10]. See Fig. 1 for a depiction of the standard participation timeline.

At RCT initiation we set out to recruit 180 participants by June 2020, allowing us 60 participants in each treatment arm. Due to unanticipated recruitment challenges, by March 2020, only 93 participants had completed (53) or were enrolled in the RCT (40), and with a newly enhanced community-based recruitment strategy (discussed later in this commentary), we expected to reach our initial recruitment goal by extending the RCT to June 2021 with a one-year no-cost funding extension. However, at the onset of the pandemic, we temporarily paused new enrollments and shifted our focus to the 40 participants who were active in various stages of the RCT at Albert Einstein College of Medicine (AECOM): 12 were completing baseline evaluations, 11 were actively receiving in-person SIT or ABA intervention, 10 were in Post 1, and 7 were in Post 2 (Table 1).

**Table 1:** Participant breakdown by study stage and randomization status at study shutdown

	Sensory integration therapy (SIT)	Applied behavior analysis (ABA)	No treatment (NT)	Not Yet Randomized	Total
**Baseline**	-	-	-	12	**12**
**Intervention**	5	6	-	-	**11**
**Post 1**	1	3	6	-	**10**
**Post 2**	4	0	3	-	**7**
**Total**	**10**	**9**	**9**	**12**	**40**

Table 1 displays the breakdown of the 40 active participants in the RCT in March 2020 when the study was initially suspended. Participants are categorized based on the treatment arm they had been assigned, or in some cases, it is indicated that participants had not yet been randomized. Participants are further categorized based on the stage of the study they were completing during the time of the initial study suspension

**Fig. 2:**
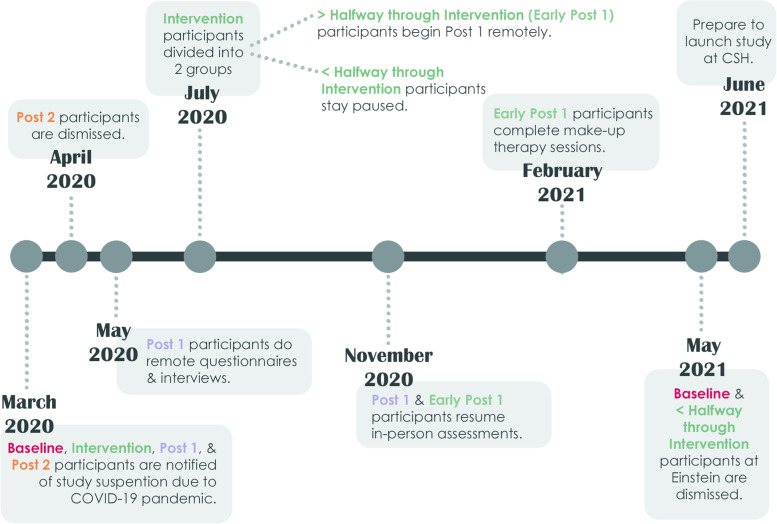
Timeline of decision-making related to COVID-19 impact

As New York City emerged as an epicenter of COVID-19 [11], our RCT, along with other nonessential research, was forced to suspend any in-person research operations until further notice. With participant safety and well-being consistently at the forefront of our decision-making, here, we discuss how we have thus far navigated the uncertainty of the pandemic and adjusted our protocol to best preserve the integrity of the RCT. We primarily focus on the RCT operations at AECOM by first describing our participant sample, then noting our response to specific challenges brought forth by the pandemic, and finally, reflecting on what this can teach us about handling unexpected interruptions to research and designing future RCTs. However, at the onset of the pandemic, we had also been preparing to open a second site for this RCT at Children’s Specialized Hospital (CSH) in New Jersey, and we later discuss how we launched recruitment at this site in the wake of COVID-19. Figure 2 offers a concise presentation of our decision-making timeline across both sites between March 2020 and June 2021.

## Participant demographics

The participants in our RCT from AECOM are representative of the diversity within our local Bronx, New York community. Approximately 60% of all participants in our current sample (*n*=93) reside in the Bronx, and an additional 10% of participants reside in Harlem and surrounding neighborhoods in Upper Manhattan. Additionally, 56% of our sample report identifying as Hispanic or Latino, which is consistent with 2021 US Census estimates for the Bronx [12]. Just over 85% of our sample is male, which is also representative of the 4:1 male-to-female ASD diagnosis ratio traditionally reported in the literature [13], albeit recent studies suggest the actual ratio may be closer to 3:1 [14]. We largely attribute our success in recruiting a representative sample to our community-based outreach strategy, which is detailed later in this commentary. Nevertheless, our goal in describing our sample’s demographics here is primarily to provide context for the reader as we detail our decision-making over the course of the pandemic throughout the remainder of the text.

## Establishing a line of communication amid the RCT suspension (March 2020)

In March 2020, in accordance with institutional policies at AECOM to pause in-person nonessential research, all 40 participants active in the RCT were directly notified of the immediate study suspension and told to await further notice regarding eventual study reopening (Fig. 2). From then on, we maintained a consistent and open dialogue with these participants via weekly text, phone, and email messages to provide updates on the state of the RCT and record any changes to participants’ usual treatment plan (including medications and external behavioral interventions). Establishing this line of communication with participants early on was critical in retaining participants throughout the study suspension and ensuring that our eventual study modifications and protocol adaptations were successfully implemented.

## Triaging participants to preserve primary outcome measures (April 2020)

By April 2020, New York City had become an epicenter of the COVID-19 pandemic, with case counts rising to roughly 5000 diagnosed cases per day, and rates of hospitalization and death were consistently highest in the Bronx and among Black and Hispanic or Latino individuals when rates were adjusted for differences in age-distributions among the groups [11]. As it became clear that the unprecedented interruption of in-person data collection would extend well into the foreseeable future, we triaged participants in a way that allowed us to focus on best preserving our RCT’s primary outcome measures in Post 1. Accordingly, our team made the decision to dismiss the seven participants that had been in Post 2 prior to study shutdown (Fig. 2). Though these participants had not fully completed all assessments typically conducted in this stage, we determined that our resources should be allocated elsewhere since we already had collected their Post 1 assessments. Upon dismissal, these participants were provided with the same packet they would have received if given the opportunity to complete the study in full, which included monetary compensation, a written summary of their assessment results, and a listing of local SIT and ABA resources.

Early dismissal of Post 2 participants allowed us to then focus our efforts on collecting datapoints from our Post 1 participants (*n*=10) as efficiently as possible, which required modifying our RCT protocol and materials to allow for remote data collection. At this point, we also continued our weekly check-ins with our Intervention (*n*=11) and Baseline participants (*n*=12), but we did not yet ask these participants to complete any remote study activities.

## Designing and implementing Post 1 remote data collection procedures (May 2020)

As previously noted, the assessment battery performed at Baseline, Post 1, and Post 2 involved a combination of neurophysiological, parent-report, and standardized clinical/OT measures, and each category of assessments lent itself to varying degrees of remote administration feasibility. For instance, neurophysiological data, in our case, EEG recordings, could not be conducted without both the participant and a research technician being present in our lab’s EEG sound-and-electrically shielded recording booth, and therefore could not be modified for remote use. On the other end of the spectrum, we found our parent-report measures more easily lent themselves to remote administration with minor adjustments. Specifically, we used videoconferencing to remotely conduct and record parent interviews that were previously conducted in person under pre-pandemic conditions. We also employed Pearson’s Q-Global® platform, a web-based system for clinical assessments and questionnaires, to send parents online questionnaires. For questionnaires that were not readily available for distribution on the Q-Global platform, we collected survey data using REDCap (Research Electronic Data Capture), which is a secure, web-based software platform designed to support data capture for research studies [15, 16]. Members of our study team coded individual questionnaire items into our RCT’s REDCap database and generated unique links for each parent to complete about their child. Through this, all questionnaires in our assessment battery could be completed securely online from any computer, smartphone, or tablet. However, our study team was also available to complete questionnaires with parents over the phone by reading all items and answer choices aloud and recording parents’ responses, though ultimately only one participant preferred to complete their questionnaires this way.

Despite our success with collecting parent-report measures online, we determined that some of the standardized clinical/OT assessments were not amenable to remote administration due to a combination of the nature of these assessments and a consideration of the resources accessible to our participant sample. For instance, the SIPT requires participants to have access to physical testing materials in order to perform 17 standardized visual, tactile, kinesthetic, and motor tasks. Conducting these assessments remotely would also require participants to have access to reliable high-speed internet, as well as ample space and appropriate testing materials to conduct assessments within their home, which was not necessarily guaranteed for all participants in our sample and not something we could provide to all our participants. Moreover, regardless of the extent to which remote assessment was feasible, we also considered that these assessments may be influenced by the different modes of administration, which raised concerns about collecting Post 1 assessments remotely when all pre-pandemic Baseline assessments had been collected in-person. As such, we opted to not pursue any remote clinical or OT assessments with our participants. Instead, we waited until we could safely resume in-person study visits to conduct any of these standardized assessments with study participants.

Of the 10 participants in Post 1 at the time of study shutdown (Table 1), a total of seven parents needed to complete remote interviews with our study team, and all did so successfully. Moreover, all 10 participants each received five online questionnaires to complete. Of the 50 total questionnaires distributed, 88% were completed successfully, with the incomplete 12% resulting from two participants who opted to discontinue their participation because of the COVID-19 pandemic.

## Re-triaging participants amid an unexpectedly prolonged RCT suspension (July 2020)

Following the success of administering questionnaires and interviews remotely for the Post 1 group and facing continued restrictions on in-person data collection, we then turned our attention to the 11 participants who were amid intervention at the point of the March 2020 RCT suspension (Table 1). Within this group, we divided participants into two categories: (1) participants less than halfway through intervention (*n*=6) and (2) participants more than halfway through intervention (*n*=5). Notably, participants in the former group had completed an average of only 2.3 therapy sessions prior to the study suspension, while those in the latter group had completed an average of 17.4 sessions at this point. We asked all participants who were more than halfway through their intervention to begin remotely completing their Post 1 questionnaires and interviews early (i.e., prior to completing all planned 30 therapy sessions of the intervention stage) (Fig. 2). We decided to skip to the Post 1 testing, as opposed to conducting remote therapy sessions to make up the remainder first, due to the inherent inconsistencies between in-person and remote therapy delivery, particularly for SIT, which participants previously conducted in a sensory gym. And, considering that these participants had already completed most of their therapy sessions in-person four months prior, the study team chose to prioritize collecting Post 1 measures as close to the pre-pandemic intervention period as possible. Understanding that participants assigned to an active treatment condition expected to receive 30 therapy sessions, these participants were offered make-up therapy sessions after they completed their participation in the Post 1. Our newly dubbed “Early Post 1” group (i.e., those more than halfway through intervention (*n*=5)) was agreeable to the changes to their participation timelines, and all participants completed their remote questionnaires and interviews successfully except for one who elected to discontinue their participation due to personal circumstances.

As “Early Post 1” participants completed remote assessments, we also developed plans for the remaining 18 participants enrolled in the RCT (Baseline participants (*n*=12) and Intervention participants less than halfway through therapy (*n*=6)). We determined that too much time would have passed between Baseline and Post 1 for the comparison to be valid and decided we would need to establish a new post-pandemic baseline for these participants before they could progress further in the RCT. Additionally, we decided that the six participants who had already been randomized into a treatment arm would not be re-randomized (i.e., they would stay in their assigned treatment arm), and once a new baseline was established, they would restart their 30 therapy sessions from zero, regardless of how many sessions they had completed pre-pandemic. However, we would not restart their study participation until we were safely able to conduct in-person assessments and confident that their participation would not be significantly interrupted by the pandemic.

## Cautiously resuming in-person study activities (November 2020)

In November 2020, we were able to resume in-person study activities in a highly limited capacity, constrained by continued physical distancing and limited occupancy guidelines (Fig. 2). Here, we again prioritized providing our remaining Post 1 (*n*=8; due to two participants dropping out) and early Post 1 (*n*=4; due to one participant dropping out) participants, who had already completed all Post 1 interviews and questionnaires remotely, with the opportunity to complete the remaining in-person Post 1 assessments. By February 2021, these participants had completed all required in-person assessments except for one participant who elected to not return for in-person study activities (Fig. 2). These participants were not asked to return for Post 2 testing, given their already significant Post 1 timeline interruption and the continued uncertainty of whether the pandemic would again disrupt future in-person data collection. As with our Post 2 participants that were dismissed in April 2020, these participants were given the same dismissal package they would have been given if they had completed the study in full. Additionally, the “Early Post 1” participants were offered the opportunity to make up any of their remaining therapy sessions with our team, and all four participants chose to do so (Fig. 2).

## Allocating RCT resources across two recruitment sites (May 2021)

In the following months, our team began preparations to restart the remaining 18 participants, but in May 2021, we made the difficult decision to instead dismiss these participants from the RCT. This decision was motivated by several reasons, but chief among them was the limited resources available to us as we moved into our second no-cost funding extension year for this study. Moreover, in the months just before the COVID-19 pandemic, our team had prepared to launch a second recruitment site at CSH in order to expand our enrollment capacity. Though restrictions on non-essential research operations at CSH had previously stalled recruitment at this site, our ability to open enrollment at CSH persuaded us to focus our efforts on study operations at one site instead of dividing our efforts across two. As such, participants at AECOM were immediately notified of our decision to not restart the study at this site, thanked for their patience throughout the pandemic, and provided with standard dismissal packets regardless of how much of the study they had completed. Again, in addition to monetary compensation, these dismissal packets included a summary of any previous testing participants completed as part of the study and information about local providers which they could explore if they were interested in continuing ABA or SIT services. Additionally, we decided to revise our original target recruitment goal from 60 participants per treatment arm to 45 participants per treatment arm in light of the COVID-19 pandemic and its challenges. We conducted an interim futility analysis with the data from all subjects who had completed post-treatment evaluation (15–20 participants per treatment arm). We calculated that this revised sample target would still have sufficient power to detect differences between the groups on at least one of our primary outcome measures and would be a more realistic goal moving forward in the wake of the COVID-19 pandemic.

## Developing a virtual recruitment strategy (June 2021)

Throughout the pandemic, before deciding against restarting enrollment at AECOM, we had continued our efforts to promote the study in preparation for an eventual restart. However, our pre-pandemic recruitment strategy had prioritized hosting and tabling at large events within the community, including autism resource fairs, parent workshops, and advocacy walks. Part of what had made this strategy successful in recruiting a representative sample for this RCT was that these large community events gave us an opportunity to interact face-to-face with community members and explain our research goals and procedures to individuals who perhaps never participated in research before or were skeptical of doing so. Members of the study team would bring study flyers, information about SIT and ABA therapies, and model EEG caps to showcase what it would be like to participate in our RCT and answer questions about the study. However, because of the prolonged COVID-19 pandemic and continued physical distancing guidelines, these large in-person events became infeasible. As such, we needed to redesign our recruitment strategy to adapt to the restrictions COVID-19 placed on in-person events.

In some instances, when previously in-person events turned to a virtual format, we worked with event organizers to host brief presentations about our research during the events so that participants would have the opportunity to still meet and hear from the study team directly. However, virtual attendance was unfortunately only a small fraction of the typical turnout for these in-person events, leading us to invest in additional recruitment avenues to complement these efforts.

One solution we developed was to forge more connections with local healthcare providers who often work with autistic children within the age range for this RCT. Though this recruitment method does not allow for the participant to interface with the study team directly the way that in-person events do, it does allow participants to learn about the study and have their questions answered from a professional they already have a relationship with. To further streamline this effort, we developed an online portal in REDCap and obtained IRB permissions to allow providers to share patient contact information (only if the patient expressed interest in the study and provided consent to this sharing) with the RCT so that the study team could follow up with the patient instead of, for example, the patient needing to remember to call the study team when they got home from an appointment with their healthcare provider. This we felt would be crucial in minimizing the steps participants needed to take to enroll in the RCT and, therefore, minimize the loss of participants during the recruitment process. Furthermore, we pursued a robust online recruitment strategy, where we used our connections with community advocacy organizations to spread the word about this RCT to the families they work with through their online newsletters and webpages. We also employed our lab’s own social media channels to promote the study by posting our IRB-approved flyers and infographics.

Though we ultimately decided to not continue enrollment at AECOM, this shift in recruitment strategy better prepared us to launch recruitment efforts at CSH, as COVID-19 restrictions on large in-person events persisted in June 2021. For recruitment at CSH, we have drawn from our efforts at AECOM and worked to establish connections with local healthcare providers within CSH’s network to connect their patients with this RCT. We also expanded our online recruitment efforts by promoting our study on social media and utilized CSH’s patient database to share information about our study. Though restrictions on in-person research activities prevented us from enrolling participants until November 2021, laying this groundwork during the initial site setup meant that we were able to generate 35+ inquiries (community members expressing interest in enrollment) in the initial weeks of our site launch. However, with the surge of the COVID-19 Omnicron variant in December 2021, we opted to enroll participants more gradually than initially planned in order to ensure that we could accommodate all participants’ time-sensitive, in-person evaluations while allowing for recommended physical distancing. As of July 2022, we have enrolled 4 participants in the RCT and we recently resumed our attendance at in-person recruitment events and continue to look for ways to connect with the local community while abiding by COVID-19 safety protocols.

## Confirming ASD diagnosis under continued mask mandates (June 2021)

Under pre-pandemic conditions, our RCT employed the Autism Diagnostic Observational Schedule, Second Edition (ADOS-2), which is a gold-standard, highly structured, ASD diagnostic tool that can quantitatively assess the severity of ASD behaviors in multiple domains [17]. In our case, the ADOS-2 was utilized to confirm ASD diagnosis at baseline, to stratify our groups based on ASD severity scores, and finally, as one of our secondary outcome measures indexing change in the severity of typical autistic behaviors because of intervention. Though the ADOS-2 had not been extensively validated as an outcome measure at the time of RCT conception [18], by collecting these data, we intended to assess ADOS-2 severity scores’ sensitivity to change, and notably, emerging research suggests these scores can serve as sensitive outcome measures [19]. Nevertheless, as a result of the prolonged COVID-19 pandemic, continued masking guidelines have prevented researchers from conducting ADOS-2 assessments, as wearing a mask during the ADOS-2 compromises its validity [20] since the examiner’s interpretation of the examinee’s organic facial expressions is integral to obtaining a severity score. We therefore sought an alternative assessment to utilize in the continuation of this RCT.

The Childhood Autism Rating Scale, Second Edition (CARS-2) [21, 22] quickly emerged as the most promising alternative to the ADOS-2 in the context of this RCT for several reasons. Most notably, administering the CARS-2 does not require interaction between a research-reliable psychologist and participant, as is the case for the ADOS-2, and does not require a set of specialized props. Instead, the CARS-2 is designed such that a psychologist observes participant behavior, and observation can be done either remotely or through a one-way observation mirror; additionally, the CARS-2-obs model allows the examiner to observe caregiver-participant interaction to identify behaviors consistent with ASD [23]. In either case, the CARS-2 allows for reliable ASD diagnostic assessment without the need for close interaction with a participant, which made it a promising solution to continuing assessment in the wake of physical-distancing guidelines. Furthermore, the CARS-2 offers cutoff scores, standard scores, and percentiles to determine ASD symptom severity, which allows us to still stratify our randomization based on ASD severity.

## Conclusions

In reflecting on our experience suspending and then gradually restarting our RCT, the importance of keeping participants at the forefront of decision-making cannot be overstated. Of course, we mean this with respect to assuring our study modifications prioritized their safety from COVID-19, but also in the sense of considering the feasibility for participants to adhere to the modifications we wished to make, and in consideration of maintaining the scientific rigor of the trial. For instance, when determining how to proceed with remote assessment, converting the entirety of our assessment protocol to a remote format would have allowed us to better preserve the intended timeline of this RCT. However, we had to consider whether our participants had sufficient access to stable internet, appropriate testing materials, and ample testing space in their homes, and this ultimately led us to virtualize our assessment battery only partially until in-person visits were safe to conduct. Therefore, for future RCTs, we would recommend considering the feasibility and validity of remote assessments when designing a testing protocol, as choosing assessments that are suitable for either in-person or remote administration would allow for greater flexibility when scheduling appointments and therefore better adherence to planned participation timelines. Greater remote participation options may also be well received by participants, who may have grown accustomed to accessing these opportunities virtually and/or prefer to continue to limit in-person activities due to continued health concerns or for the sake of convenience.

Furthermore, maintaining a consistent dialogue with all active participants and triaging participants to best preserve our primary outcome measures was essential to navigating the dynamic unfolding of the COVID-19 pandemic. Participants were contacted weekly by the study team to check in with families and provide updates on the status of the RCT. In doing so, we were able to accurately record any changes to participants’ treatment plans outside of the RCT, ensure timely completion of remote assessments, and eventually, coordinate in-person assessments safely and efficiently. And, having an open line of communication with participants allowed us to keep careful note of variables that were beyond our control, including whether participants were attending school virtually, in-person, or a mix of the two. As such, we believe that effective triaging and communication are key to preserving the most critical elements of RCTs in the face of unexpected interruptions to research.

The pandemic sparked unprecedented interruption to daily life on a worldwide scale that one hopes is a once-in-a-century event. Nevertheless, this experience makes clear that assuming the possibility of such disruptions to life, and therefore clinical research, again, would be wise. Indeed, clinical trials have always been and will continue to be at risk for major or minor interruptions, whether it be from extreme weather events, participants’ schedules, or other unforeseen circumstances [24]. This is particularly the case when considering RCTs such as ours, which originally relied solely on in-person appointments over an extended timeframe (6 to 9 months). In documenting how our RCT navigated the COVID-19 pandemic, our goal is to provide a recommendations (Table 2) within which to plan for these potential events while conceptualizing future RCTs and managing unexpected interruptions to research.

**Table 2:** Summary of recommendations for future RCT design and managing unexpected interruptions to research

• When designing future RCTs, we suggest choosing assessments with virtual-adaptability whenever possible, making specific note not only of the validity of these assessments when conducted remotely, but also their practical feasibility given ones’s participant population.	
• When faced with unexpected interruptions to research, we further stress the importance of triaging paticipants to best preserve an RCTs primary outcome measures, while still honoring commitments to participants and keeping an open dialogue with them such that their voices can continue to guide decision-making.	

## Data Availability

Data sharing is not applicable to this article as no datasets were analyzed during the current study and data collection is ongoing.

## Abbreviations

ABA: Applied behavior analysis
ADOS-2: Autism Diagnostic Observation Schedule, Second Edition
AECOM: Albert Einstein College of Medicine
ASD: Autism spectrum disorder
CARS-2: Childhood Autism Rating Scale, Second Edition
CSH: Children’s Specialized Hospital
EEG: Electroencephalogram
IRB: Institutional Review Board
NT: No treatment
OT: Occupational Therapy
RCT: Randomized control trial
REDCap: Research Electronic Data Capture
SIPT: Sensory Integration and Praxis Test
SIT: Occupational Therapy using Ayres Sensory Integration® Therapy
SPM: Sensory Processing Measure
WASI-II: Wechsler Abbreviated Scale of Intelligence–Second Edition
